# Detection of Circulating Tumor Cell Subpopulations in Patients with Head and Neck Squamous Cell Carcinoma (HNSCC)

**DOI:** 10.1371/journal.pone.0113706

**Published:** 2014-12-05

**Authors:** Patrick Weller, Ivonne Nel, Philipp Hassenkamp, Thomas Gauler, Anke Schlueter, Stephan Lang, Paulette Dountsop, Andreas-Claudius Hoffmann, Götz Lehnerdt

**Affiliations:** 1 Department of Otorhinolaryngology, University Hospital Essen, University of Duisburg-Essen, Essen, Germany; 2 Molecular Oncology Risk-Profile Evaluation, Department of Medical Oncology, West German Cancer Center, University Duisburg-Essen, Essen, Germany; 3 Department of Otorhinolaryngology, Evangelisches Krankenhaus Duesseldorf, Duesseldorf, Germany; 4 Department of Otorhinolaryngology, St. Anna-Klinik, Wuppertal, Germany; Virginia Commonwealth University, United States of America

## Abstract

**Background:**

Since image based diagnostic tools fail to detect early metastasis in head and neck squamous cell carcinoma (HNSCC) it is crucial to develop minimal invasive diagnostic methods. A promising approach is to identify and characterize circulating tumor cells (CTC) in the peripheral blood of HNSCC patients. In this pilot study, we assessed which non-hematopoietic cell types are identifiable and whether their numbers differ in pre- and postoperative blood samples.

**Methods:**

20 ml citrated peripheral blood was taken from 10 HNSCC patients before and after curative resection. CTC were enriched using density gradient centrifugation. CTC presence was verified by multi-immunofluorescence staining against cytokeratin (CK; epithelial), N-cadherin (mesenchymal); CD133 (stem-cell), CD45 (hematopoietic) and DAPI (nucleus). Individual cell type profiles were analyzed.

**Results:**

We were able to detect cells with epithelial properties like CK+/N-cadherin−/CD45− and CK+/CD133−/CD45− as well as cells with mesenchymal features such as N-cadherin+/CK−/CD45− and cells with both characteristics like N-cadherin+/CK+/CD45−. We also observed cells showing stem cell-like features like CD133+/CK−/CD45− and cells with both epithelial and stem cell-like features such as CD133+/CK+/CD45−. The number of CK positive cells (p = 0.002), N-cadherin positive cells (p = 0.002) and CD133 positive cells (p = 0.01) decreased significantly after resection. Kaplan-Meier test showed that the survival was significantly shorter when N-cadherin+ cells were present after resection (p = 0.04; 474 vs. 235 days; [HR] = 3.1).

**Conclusions:**

This is - to the best of our knowledge- the first pilot study identifying different CTC populations in peripheral blood of HNSCC patients and showing that these individual cell type profiles may have distinct clinical implications.

## Introduction

Since image based diagnostic tools fail to detect early metastasis in HNSCC it is crucial to develop minimal invasive diagnostic methods to characterize entities and to find markers that could help choosing the appropriate treatment and monitoring response at an early stage.

Circulating tumor cells (CTC) could serve as a “liquid biopsy” for individualizing and monitoring therapy in patients with solid tumors [1], [2]. So far, CTC detection methods consisted of enrichment and subsequent identification mostly with anti-cytokeratin (CK) or epithelial cell adhesion molecule (EpCAM) antibodies. CK-positive cells are thought to be absent or to be present in the blood of healthy subjects in very low numbers [3]. CTC have extensively been described in breast and lung cancer and EpCAM-positive CTC quantification has been linked to patient outcome [4]–[7]. Up to now only a few reports have been published on the isolation of CTC in HNSCC [8]–[15].

Standardized approaches with currently available enrichment and detection techniques are based on physical or biological properties of CTC and challenged by their cellular heterogeneity and plasticity. Epithelial-to-mesenchymal transition (EMT) can cause alteration of cellular features and loss of epithelial properties leading to a partial or complete switch to a mesenchymal phenotype. Particularly stem cells have the ability to take on characteristics of other cell types [16].

We recently developed a CTC detection method based on multi-parameter immunofluorescence microscopy (MPIM) that includes but is not solely dependent on epithelial markers such as CK or EpCAM and also detects cells with mesenchymal and stem cell-like characteristics [17]. We were able to show that the individual composition of these CTC profiles correlated to therapeutic success in hepatocellular carcinoma, non-small cell lung cancer and renal cell carcinoma [17]–[19]. In this preliminary study, we used a slightly modified methodology including density gradient centrifugation but no depletion of CD45-positive cells and addressed the question whether different types of CTC are identifiable in the peripheral blood of patients with HNSCC and, if so, whether their distribution may serve as a predictor of treatment response or outcome. With this approach we wanted to scrutinize whether developing a blood-based multi-marker panel for personalized treatment of HNSCC is warranted.

## Materials and Methods

### Ethics Statement and Study Population

Written informed consent was obtained from all patients before participating in the study. Blood sample collection and analyses were approved by the Review Board of the Ethics Committee of the regional Medical Association Nordrhein; Germany (2012304) and the Medical Department, University of Essen-Duisburg; Germany (12-5047-BO). We analyzed 10 patients with HNSCC before and after curative surgical resection. The clinico-pathological characteristics of the patients are listed in Table 1. Tumor staging was performed according to the criteria of the TNM Classification by the Union for International Cancer Control (UICC) [20].

**Table 1:** Patients Demographics.

	Patients (n = 10)	
Demographic	No.	%
**Tumor Staging**		
T1	1	10
T2	3	30
T3	3	30
T4	2	20
Tx	1	10
**Lymph nodes**		
N0	5	50
N2	4	40
N3	1	10
**Grading**		
G2	10	100
**Age**		
Median, years	60	
Range	56–76	
**Gender**		
male	8	80
female	2	20
**Smoking**		
Yes	10	100
**Alkohol**		
yes	8	80
no	2	20
**Localization**		
Larynx	7	70
Oropharynx	2	20
Oral cavity	1	10

G2: moderately differentiated.

### Preparation of blood samples and CTC enrichment

**Figure 1:**
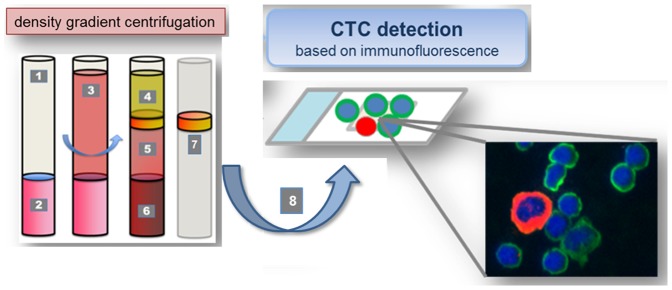
Basic principle of CTC isolation. 1. Leucosep tube: 2. Separation media; 3. Whole blood/PBMNC mixture; 4. Plasma; 5. Separation media after centrifugation; 6. Erythocytes; 7. Buffy coat incl. CTCs; Cell suspension containing CTCs for Cellspin and multi-immunofluorescence staining. Before and after curative resection, mononuclear cells and CTC were isolated from peripheral venous blood using density gradient centrifugation. Cell suspensions were carefully spun onto glass slides. The presence of CTC was verified by immunofluorescence staining against pan-CK (epithelial), N-cadherin (mesenchymal), CD133 (stemcell-like); counterstaining with CD45 (hematopoietic) and DAPI (nucleus). CTC and hematopoietic cells were enumerated, normalized and individual cell type profiles were analyzed and correlated to therapeutic outcome in 10 patients.

20 ml citrated peripheral venous blood were drawn from HNSCC patients directly before and immediately after curative tumor resection and processed within 24 h after collection. Blood sample preparation was done as described previously [17], [19]. Briefly, 20 ml of blood were diluted with 10 ml PBS and carefully layered into a Leucosep tube containing 16 ml Ficoll-Paque (GE-Healthcare) below a porous barrier. After buoyant density gradient centrifugation (1600×g, 20°C, 20 min) the interphase consisting of peripheral blood mononuclear cells (PBMNC) and CTC was removed and washed. The cell suspension was spun onto 2–4 glass slides per sample containing a maximum of 6×10^6^ cells per slide using the Cell Spin II centrifuge (Tharmac, Waldsolms, Germany), air-dried and subsequently fixated with 96% Ethanol. Slides were stored at 4°C until subjected to immunocytochemical staining (Fig. 1). The presence of CTC was verified by multi-immunofluorescence staining against pan-CK (epithelial), N-cadherin (mesenchymal), CD133 (stem-cell-like), counterstaining with CD45 (hematopoietic) and DAPI (nucleus). CTC and hematopoietic cells were enumerated, normalized and expressed as number of CTC per 1000 PBMNC. Individual cell type profiles were analyzed and correlated to therapeutic outcome in 10 patients.

### Identification of CTC subtypes using multi-immunofluorescence staining

Immunofluorescence staining of epithelial, mesenchymal, stem cell-like and hematopoietic cells was carried out as described previously [17]. Briefly, the staining method included fixation of the cells in 4.5% paraformaldehyde for 15 min, washing in PBS, permeabilization with 1× Perm/Wash Buffer (BD Biosciences, Franklin Lakes, USA) for 10 min, washing in PBS, blocking of unspecific antibody reactions by incubation with blocking solution containing 5% BSA for 30 min, binding of primary antibodies (final concentration: 5 µg/ml, Table 2) either anti-pan-CK and anti-N-cadherin (or anti-CD133 for CTC and anti-CD45 for hematologic cells overnight at 4°C, wash in 0,1% Tween, binding of secondary antibodies (Table 2) for 30 min at 37°C, washing in 0,1% Tween. Subsequently, cells were stained with 4′6-Diamidino-2-phenylindole dihydrochloride (DAPI; Sigma-Aldrich, St. Louis, MO) for 10 min, mounted with anti-fading medium (Invitrogen) and stored in the dark until evaluation. As described previously, for each test a control slide with a mixture of PBMNC (CD45-positive, pan-CK-negative) from a healthy donor spiked with different cell lines (epithelial, mesenchymal, CD133 positive cells) were used as positive control. Microscopic evaluation was carried out using the digital Keyence BZ9000 (Biorevo, Osaka, Japan) all-in-one fluorescence microscope with integrated camera and BZ-Analyzer Software. We used pseudo colors to depict cells. Stained slides were manually examined and CTC were detected within the same areas, each consisting of 10 visual fields using a 20× magnification on both slides. Samples from 12 healthy donors were processed and examined under the same conditions in order to define cut-off values for false-positive events.

**Table 2:** Antibodies.

Primary Antibodies (anti-human)				
CD45 (MEM-28)	mouse	monoclonal	ab8216	Abcam, Cambridge, UK
N-cadherin (EPR1792Y)	rabbit	monoclonal	2019-1	Epitomics, Burlingame, CA
CD133	rabbit	polyclonal	orb18124,	biorbyt, Cambridge, UK
Pan-CK	guinea pig	polyclonal	ABIN126062	antibodies-online, Atlanta, GA
**Secondary Antobodies**				
Cy3-conjugated AffiniPure	goat anti-mouse	Jackson Immuno Research, Hamburg, Germany		
FITC-conjugated AffiniPure	goat anti-rabbit	Jackson Immuno Research, Hamburg, Germany		
AlexaFlour647-conjugated AffiniPure F(ab′)2 Fragment	goat anti-guinea pig	Jackson Immuno Research, Hamburg, Germany		

### Statistical Analysis

Statistical tests were performed according to previously published studies by our group [17], [21], [22]. The associations among CTC subtypes and clinico-pathological parameters were tested with Spearman test for bivariate correlations. Wilcoxon test for paired samples was used to compare differences of various factors at different time points. Mann-Whitney test for independent samples was used to compare differences of various factors in distinct subgroups. Survival curves (500-days cutoff) were plotted according to the Kaplan–Meier method to test correlations of overall survival with CTC subtypes. Significance was tested using the log-rank test. The level of significance was set to P<0.05. All P values were based on two-sided tests. All statistical analyses were performed using the Software Packages SPSS for Windows (Version 19.0; SPSS Inc., Chicago, IL) and Medcalc, Version 12.3.0 (Mariakerke, Belgium).

## Results

### Immunofluorescence based identification of CTC subtypes

**Figure 2:**
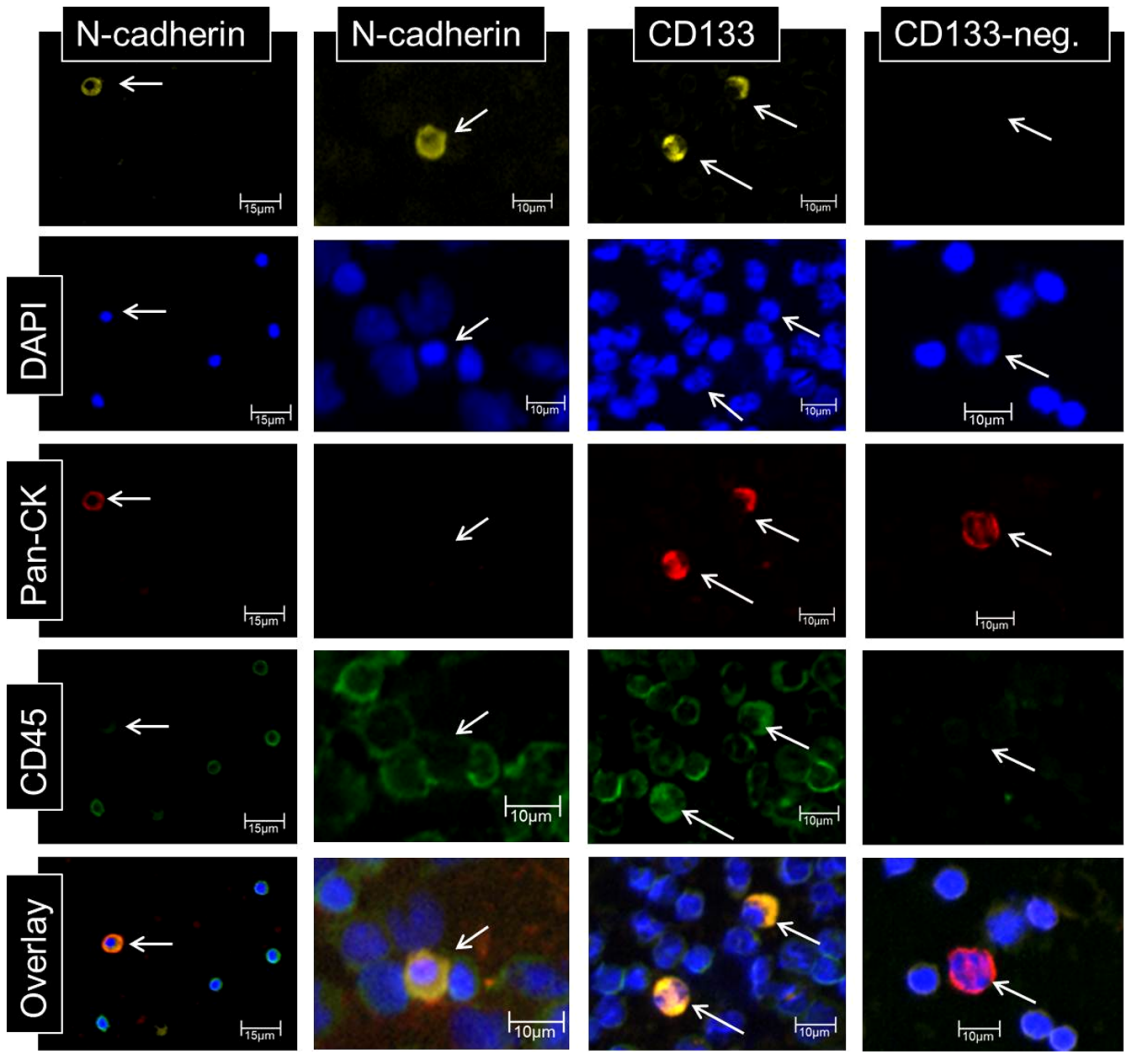
Selection of detected CTC subtypes using multi-immunofluorescence staining. As described above we detected a variety of CTC subtypes. N-cadherin and CD133 were stained on separate slides, respectively, together with CK, CD45 and DAPI. Pseudo colours were used to depict CTC subtypes: N-cadherin (mesenchymal,yellow); CD133 (stem-cell-like, yellow), pan-cytokeratin (CK; epithelial, red), CD45 (hematopoietic, green) and DAPI (nucleus, blue). We were able to detect cells with epithelial and mesenchymal properties like N-cad+/CK+/CD45low, as well as cells with mesenchymal features such as N-cad+/CK−/CD45low. We also observed cells showing mixed characteristics like CD133+/CK+/CD45+ and cells with epithelial characteristics such as CD133−/CK+/CD45− cells. More CTC subtypes were detected, but are not shown in this figure.

For the investigation of cellular subtypes a multi-staining method was required in order to detect various epithelial, mesenchymal, stem cell-like and hematopoietic characteristics. Therefore, we used multi-immunofluorescence staining for CTC-subtype detection. In HNSCC blood samples we observed cells with mesenchymal features such as N-cadherin+/CK−/CD45− and cells with epithelial properties like CK+/N-cadherin−/CD45− and CK+/CD133−/CD45− and cells with both characteristics like CK+/N-cadherin+/CD45−. We also detected cells that stained positive for CD133 which is believed to be a cancer stem cell marker and found subtypes such as CD133+/CK−/CD45− and CD133+/CK+/CD45− cells. In addition, we found cells that stained positive for potential markers of CTC and CD45 such as N-cadherin−/CK+/CD45low, N-cadherin+/CK−/CD45low N-cadherin+/CK+/CD45low as well as CD133+/CK+/CD45+, CD133−/CK+/CD45low and CD133+/CK−/CD45lowcells (**Fig. 2**). CTC were enumerated and CTC profiles of each patient were examined. We scored the total amount of N-cadherin-positive, CK-positive and CD133-positive cells, and calculated a ratio of mesenchymal to epithelial cells and stem cell-like to epithelial cells, respectively, after density gradient centrifugation. We normalized the enumerated potential CTC against the total PBMNC number detected in the DAPI channel in each visual field and calculated the number of CTC per 1000 PBMNC. Analysis of samples from healthy donors revealed the following cut-off values for false positive events per 1000 PBMNC: 0.07 CD133+/CK+ cells; 0.07 CD133+/CK− cells; 0.01 N-cadherin+/CK+ cells; 0.03 N-cadherin+/CK− cells and a total of 0.6 CK+ cells.

### CTC-subtypes and clinical outcome

**Figure 3:**
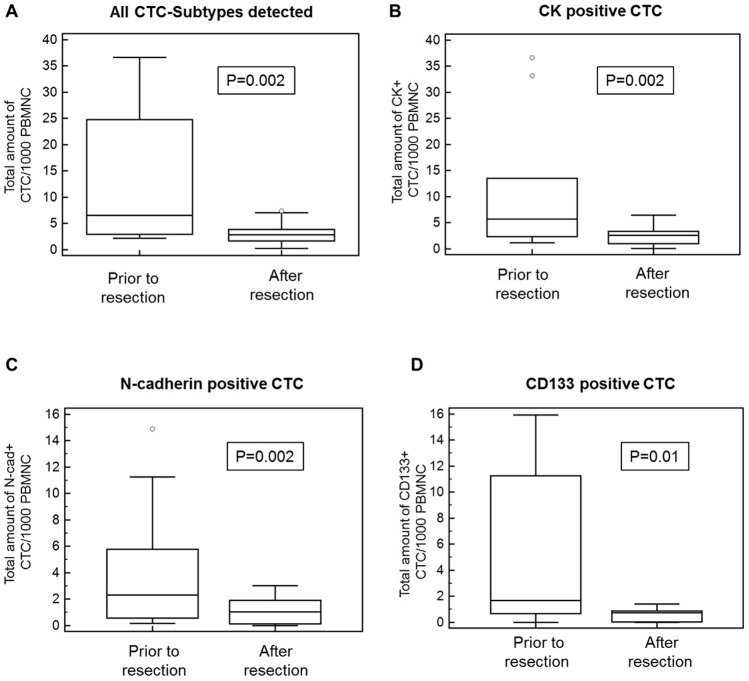
Subtype analysis revealed a significant decrease of epithelial CK+, mesenchymal N-cadherin+ and stem cell-like CD133+ cells after resection. **A**) Wilcoxon test showed that the total amount of CTC/1000 PBMNC was significantly decreased after resection (p = 0.002). CTC-Subtype analyses revealed that the total amount of epithelial (CK-positive; **B**), mesenchymal (N-cadherin-positive; **C**) and stem cell-like (CD133-positive; **D**) CTC, respectively, was significantly decreased after resection.

**Figure 4:**
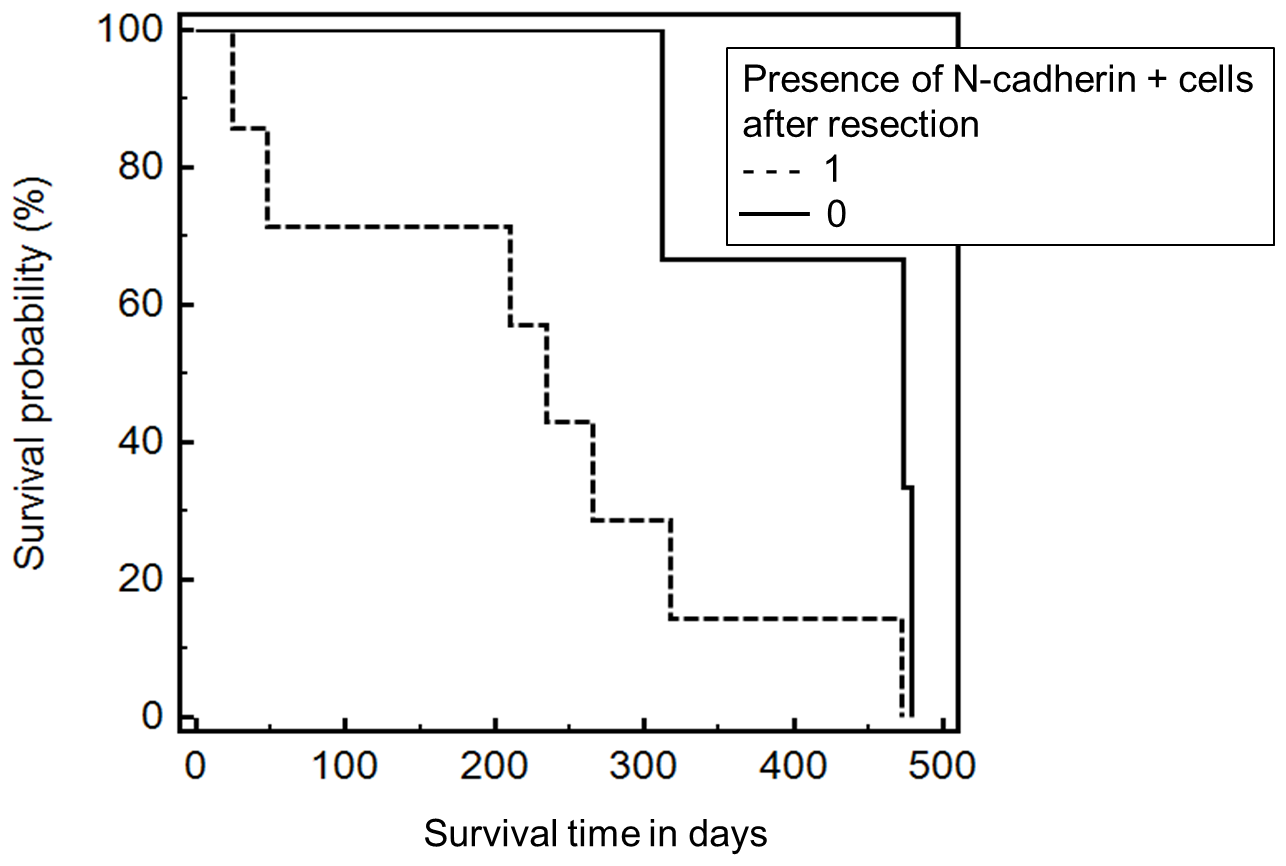
Kaplan Meier test showed a significantly shortened survival when N-cadherin+ cells were present after resection (p = 0.04; 474 vs 235 days; CI: 0.8856–10.8339; [HR] = 3.1).

**Figure 5:**
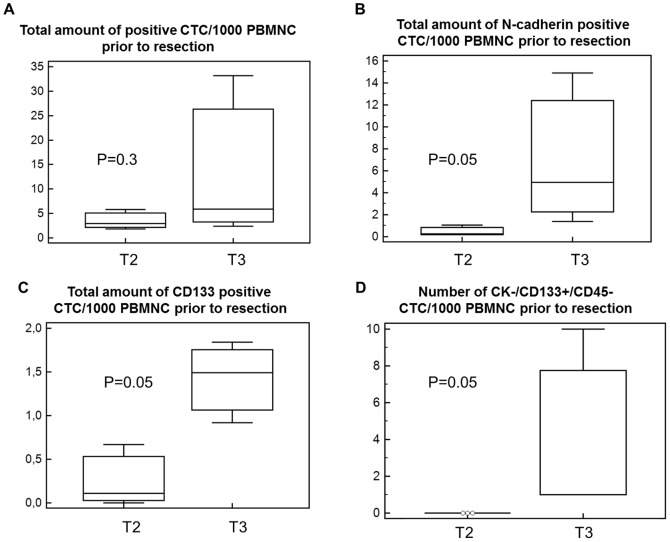
Association between CTC subtypes and tumor staging. **A**) Mann-Whitney test showed that he total amount of CTC was elevated in T3 patients compared to T2, but the difference did not reach level of significance. **B–C**) Prior to resection, the amount of N-cadherin-positive (mesenchymal) and CD133-positive (stem cell-like) CTC was significantly higher in stage T3 compared to T2 patients (both p = 0.05). **D**) Stem cell-like CK−/CD133+/CD45− cells were only present in stage T3 patients.

Spearman's rank correlation revealed that the survival time was significantly associated to the presence of N-cadherin+ (p = 0.04) and CK+ cells (p = 0.06) and the total amount of N-cadherin+ cells (p = 0.04) and CK+ cells (p = 0.05). Survival was also significantly correlated with the number of CK+/N-cadherin+/CD45− (p = 0.04), CK+/N-cadherin+/CD45+ (0.03) and CK−/CD133+/CD45+ cells (p = 0.03). Furthermore, Spearman test showed statistical association between survival and the number of N-cadherin+/CK−/CD45− (p = 0.17), N-cadherin+/CK+/CD45+ (p = 0.13) and N-cadherin−/CK+/CD45+ (p = 0.14) cells as well as the ratio of mesenchymal to stem cell-like cells (N-cadherin/CD133; p = 0.15). Interestingly, tumor staging was significantly associated to the number of stem cell-like CD133+/CK−/CD45− (p = 0.004), CD133+/CK+/CD45+ (p = 0.01) cells and the ratio of epithelial to mesenchymal cells (CK/N-cadherin; p = 0.01). The total number of CD133+ cells was significantly correlated with epithelial CK+/N-cadherin−/CD45− (p = 0.01), CK+/CD133−/CD45− (p = 0.02) and mesenchymal N-cadherin+/CK−/CD45− cells (p = 0.03). Wilcoxon test for paired samples showed that the total number of CTC was significantly decreased after resection compared to CTC prior to resection (p = 0.002). The subtype analysis using Wilcoxon test revealed a significant decrease of epithelial CK+ (P = 0.02), mesenchymal N-cadherin+ (p = 0.02) and stem cell-like CD133+ cells (p = 0.01) after resection (Figure 3 A–D; Table 3). Kaplan Meier test indicated a significantly shortened survival time when N-cadherin+ cells were present after resection (p = 0.04; 474 vs 235 days; CI: 0.8856–10.8339; [HR] = 3.1; Figure 4). To investigate the association between CTC subtypes and tumor staging we used Mann-Whitney test and revealed that the total number of CTC and the number of epithelial CK+ CTC was elevated in stage III compared to stage II patients, but did not differ significantly (both p = 0.3; Fig. 5 A). Interestingly, the number of mesenchymal N-Cadherin+ (p = 0.05) and stem cell-like CD133+ (p = 0.05) cells as well as CK−/CD133+/CD45− cells (p = 0.05) was significantly increased in Stage III compared to stage II patients (Figure 5 B–D; Table 4).

**Table 3:** CTC Quantification.

	Range	Median
**Total amount of positive CTC/1000 PBMNC**		
prior to resection	2–37	7
after resection	0–7	3
**Total CK+ cells/1000 PBMNC**		
prior to resection	1–37	6
after resection	0–6	3
**Total CD133+ cells/1000 PBMNC**		
prior to resection	0–16	2
after resection	0–1	1
**Total N-cadherin+ cells/1000 PBMNC**		
prior to resection	0–15	2
after resection	0–3	1

**Table 4:** Clinical Data and CTC Subtypes.

Patient Nr.	Tumor Stage	Lymph Nodes	Localization	Total CTC*		CK+ CTC*		N-cadherin+ CTC*		CD133+ CTC*	
				Before**	After**	Before**	After**	Before**	After**	Before**	After**
1	T2	N0	Larynx	5,83	3,4	5,83	3,34	0,25	0,13	0,67	1,11
2	T4a	N0	Larynx	17,06	1,47	1,14	0,06	0,57	0	15,92	1,41
3	T1a	N0	Larynx	36,67	3,86	36,61	3,21	5,78	1,93	11,56	0,8
4	T4a	N0	Larynx	24,77	7,39	13,51	5,39	11,26	1,2	11,26	5,19
5	Tx	N3	Oropharynx	5,62	2,05	5,62	2,05	3,24	1,3	2,37	0,76
6	T3	N2c	Larynx	2,38	0,24	2,38	0,2	1,38	0,07	0,92	0,03
7	T3	N2b	Larynx	7,21	3,55	5,88	2,89	4,93	2,39	1,84	0,74
8	T2	N2b	Oral Cavity	2,16	1,67	1,86	0,99	1,04	0,9	0	0,05
9	T2	N0	Oropharynx	2,92	2,35	2,92	2,35	0,18	0,12	0,11	0
10	T3	N2c	Larynx	34,33	7,05	33,18	6,47	14,9	3,01	1,49	0,58

* per 1000 PBMNC;

** refers to surcical resection.

## Discussion

Within this study we examined the individual CTC composition prior to and after curative resection in patients with HNSCC. Multi-immunofluorescence based morphological analysis revealed a variety of CTC subtypes with epithelial, mesenchymal, stem cell-like or mixed characteristics such as N-cadherin+/CK−/CD45−; N-cadherin−/CK+/CD45−; CD133+/CK−/CD45− and CD133−/CK+/CD45− cells. Analyses of individual CTC profiles indicated that the presence as well as the number of mesenchymal N-cadherin-positive and epithelial CK-positive CTC was associated to shortened survival. The presence of stem cell-like CD133-positive cells was correlated to tumor staging. The total number of CTC and detected CTC subtypes were significantly decreased after resection. Kaplan Meier test confirmed that the survival time was significantly shorter when N-cadherin-positive cells were detected after surgery, implying it as marker of poor prognosis. Our data are in line with a study by Nguyen *et al.* who examined the expression of N- cadherin using immunohistochemistry [8]. They observed high N-cadherin expression in 52 of 80 paraffin-embedded HNSCC cases and reported a significant correlation with malignant behaviors. Subsequently, they suggest N-cadherin to play an important role in malignant behaviors of HNSCC and that cadherin switching might be considered as a discrete critical event in EMT and metastatic potential of HNSCC.

Up to now, only few reports have been published on CTC in HNSCC patients. Not long ago, Jatana *et al.* presented a purely negative depletion process using erythrocyte lysis followed by immunomagnetic labeling with CD45-antibodies and immunocytochemical staining against CD45, CK and DAPI to separate and quantify CTC in HNSCC [9]. Subsequently, Balasubramanian *et al.* used this negative enrichment method and reported the presence of CTC with mesenchymal and stem cell-like characteristics such as vimentin, EGFR, N-cadherin and CD44 [10]. They emphasized the existence of CTC phenotypes suggesting EMT and described that the presence of CTC in HNSCC correlated with worse disease-free survival. Their initial results add support to our findings that CTC subpopulations with mesenchymal characteristics are not only detectable but also associated to worse clinical outcome in patients with HNSCC when present after surgical resection. A study by Winter *et al.* showed, for the first time, that almost all patients with advanced HNSCC have circulating cells at the time of surgery [11]. They applied immunomagnetic enrichment and RT-PCR detection of CTC using four epithelial markers (ELF3, CK19, EGFR and EphB4). In a pilot study with samples from 16 patients the CTC detection was not associated with significant differences in overall or disease free survival though. Another study analyzing epithelial EpCAM+/CK+/CD45− CTC using flow cytometry followed by RT-PCR was performed by Hristozova *et al.* [12]. They reported no significant association of CTC presence to T stage. Here we are able to confirm that in our study the total amount of detected CTC prior to surgery did not show significant association to tumor staging. However, the number of mesenchymal and stem cell-like CTC subtypes was significantly increased in T3 compared to T2 patients prior to resection. Nichols *et al.* used the CellSearch system and reported that only 6 out of 15 patients (40%) had detectable CTC [13]. Also Buglione *et al.* applied the CellSerach system and found that a decreased CTC number or their absence throughout the treatment seemed related with non-progressive disease [14]. The latest study using the CellSearch system was published by Bozec *et al.* who evaluated the potential detection of CTC in patients with locally advanced HNSCC and intended to identify the clinical factors predictive of CTC presence [15]. They detected CTC in only 8 of 49 patients (16%) before therapy. In contrast to the CellSearch based studies, our findings strengthen the hypothesis that CTC subtype analysis including mesenchymal and stem cell-like properties might reveal more detailed clinical association than epithelial markers alone. EMT-type cells and cancer stem cells (CSC) are believed to play critical roles in cancer metastasis and drug resistance. The formation of CSC and the event of EMT is a dynamic process which is triggered by the interaction of various cellular signaling pathways such as Hedgehog, Notch, PDGF, Wnt, TGF-β, Akt, and NF-κ B signaling pathways [23]. Recently the existence of circulating mesenchymal stem cells (MSC) derived from peripheral blood was reported [24]–[26]. It was described that MSC have the ability to migrate from bone marrow to damaged tissue via the circulating peripheral blood to promote regeneration [27]. This process may involve hyper stimulation of bone marrow production using granulocyte colony stimulating factor (G-CSF) resulting in the occurrence of a mixture of MSC, hematopoetic stem cells and other immature progenitor cells [25], [28], [29]. Furthermore, the literature revealed that MSC migrate to and proliferate within tumor sites [30].

In this study we were able to observe a distinct proportion of cells that stained positive for pan-CK and CD45, a phenomenon already described by Yu and colleagues [31]. The additional CD45+ staining may not be exclusive for hematopoietic cells, but may hypothetically be acquired during the dormant state in the bone marrow or through effects comparable to trogocytosis, i.e. transfer of membrane proteins.

Taken together, our results support the hypothesis that the examination of individual CTC profiles might be used as a follow up measure in addition to clinical data during treatment and to develop novel targeted therapeutic options in HNSCC.
